# GLUQ: Global-Local Representation Learning for UHD Image Quality Assessment

**DOI:** 10.3390/e28080861

**Published:** 2026-08-01

**Authors:** Bing Zhu, Enqi Chen, Ming Huang, Xuemin Ren, Shaode Yu, Qiurui Sun

**Affiliations:** 1 School of Information and Communication Engineering, Communication University of China, Beijing 100024, China; zhubing1218@cuc.edu.cn (B.Z.); 202311043037@mails.cuc.edu.cn (E.C.); 202311043023@cuc.edu.cn (M.H.); 202520085410002@mails.cuc.edu.cn (X.R.); 2 Center of Information & Network Technology, Beijing Normal University, Beijing 100875, China

**Keywords:** Ultra-High-Definition Image, image quality assessment, global-local representation, graph representation learning, graph convolutional network

## Abstract

Ultra-high-definition (UHD) blind image quality assessment (BIQA) is challenging because native-resolution inference is computationally expensive, whereas common strategies of resizing or patching may suppress scale-sensitive distortions and weaken the relationship between local artifacts and global scene context. To address this challenge, a Global-Local graph representation learning framework for UHD image Quality prediction (GLUQ) is proposed, which models structural dependencies among patches rather than treating them as independent views. Specifically, it samples aspect ratio-aligned patches from each UHD image, encodes these patches as graph nodes and constructs a hybrid *k*-nearest-neighbor graph via weighted spatial proximity and feature similarity. Residual graph convolution is used to propagate contextual information across regions, and gated attention pooling is used to aggregate patch-level evidence into image-level quality prediction. Besides, an exponential moving average normalized multi-objective loss function is adopted to stabilize the joint optimization of regression, correlation, and ranking objectives. Experiments on the UHD-IQA benchmark database show that GLUQ achieves the lowest RMSE among the compared methods with competitive PLCC and SRCC, indicating strong absolute-score calibration for UHD image quality prediction. The results suggest modeling global-local graph relations enhances quality prediction for UHD images with extremely high-resolution visual content.

## 1. Introduction

BIQA aims to quantify perceptual image quality without access to pristine references. It plays an important role in image acquisition, data compression, signal transmission, quality enhancement, and visual monitoring [1]. In contrast to full-reference methods [2], BIQA is more practical for real-life applications. This reference-free setting makes BIQA a key technique for perceptual optimization in visual communication [3].

The development of BIQA approaches has progressed through several stages. Firstly, BIQA mainly relied on natural scene descriptors under the assumptions or observations that distortions perturb regular statistical patterns of natural images [4,5,6], while support vector regression and other models build the relationships between quantitative features and image quality scores [5,6,7,8]. Later, patch-based deep networks improved prediction accuracy by learning quality-aware representations in an end-to-end manner from training samples, thereby eliminating the need for feature handcrafting [9,10,11,12,13]. After that, Transformer-based methods have strengthened long-range context modeling and flexible input handling, and large-scale pre-training on diverse databases has improved representation quality [14,15,16]. Beyond backbone changes, recent algorithms begin to absorb ideas from reinforcement learning and vision-language models that increasingly treat the BIQA task as a richer semantic and perceptual reasoning problem [17,18,19,20].

With the rapid advancement of imaging sensors and display technologies, UHD images have become increasingly prevalent in consumer and professional applications [21]. Benefiting from rich visual details and high spatial fidelity, UHD images support more immersive visual experiences, more reliable machine perception, and higher-quality content production. However, the enormous resolution of UHD images presents substantial challenges for quality assurance [22]. Full-resolution processing is computationally prohibitive, and common strategies, such as *simple resizing* and *independent patching*, are adopted. *Simple resizing* compresses the entire UHD image into a much lower resolution that keeps global scene semantics, lowers computational costs, and facilitates the deployment of existing BIQA models without major architectural redesign [22,23,24]. However, it also inevitably introduces substantial information loss by compressing millions of pixels into a fixed-size representation, and thereby, fine-grained local distortions and high-frequency quality cues are significantly weakened or even removed, limiting the model’s ability to accurately assess perceptual quality in UHD images [23]. *Independent patching* preserves native-resolution details by processing a limited number of local patches that retains fine-grained distortion details, enables patch-level quality representation learning, supports multi-scale analysis, and allows existing pre-trained models to be readily adopted [9,10]. However, by decomposing an image into numerous local patches, *independent patching* inevitably sacrifices global contextual information and often fails to capture the global content structure and long-range contextual dependencies due to their restricted spatial coverage. Moreover, important distortion regions may be overlooked during patch sampling, leading to unstable quality predictions. Therefore, the common strategies may sacrifice perceptually important information by suppressing scale-sensitive distortions and disrupting the relationship between global context and local artifacts [23,24].

The Ultra-High-Definition Image Quality Assessment (UHD-IQA) benchmark database and the Advances in Image Manipulation 2024 UHD blind photo quality assessment Challenge (referred to as Challenge hereafter) further establish that directly adapting conventional BIQA models to 4 K imagery is difficult under both perceptual and efficiency constraints [21,22,23,24,25]. Representative Challenge methods, such as UIQA [24], combine global aesthetics, local distortion fragments, and saliency-guided cues for UHD quality prediction, whereas a pseudo-reference dual-branch route based on super-resolution reconstruction has also been explored [26]. In parallel, graph-based and relation-aware BIQA methods have shown the value of modeling distortion relations, non-local dependencies, adaptive graph attention, or inter-patch interactions [27,28,29,30].

In this study, we propose the framework named GLUQ that bridges patch-level local evidence and image-level perceptual quality prediction. Unlike *resizing*-based methods, which sacrifice high-frequency distortion information for computational efficiency, and *patching*-based methods, which preserve local details at the expense of global contextual awareness, GLUQ provides a balanced solution by jointly preserving native-resolution patch characteristics while explicitly modeling their spatial and semantic relationships. The advantages of GLUQ are twofold. Firstly, it partitions each UHD image into native-resolution patches and uses graph representation learning (GRL) to model inter-patch dependencies. This design preserves fine-grained distortions without introducing resampling artifacts, while simultaneously capturing both local quality cues and global contextual structure. As a result, GLUQ produces a more comprehensive and perceptually consistent quality representation for UHD images, effectively bridging the gap between *resizing*-based global modeling and *patching*-based local modeling. Secondly, GLUQ enables information exchange among geographically and semantically related regions, allowing the model to capture both localized distortion patterns and holistic scene structures. Such a design is suitable for UHD images, where perceptual quality is jointly determined by fine-grained local artifacts and large-scale contextual information. Consequently, GLUQ delivers more reliable and human-consistent quality prediction while maintaining favorable computational efficiency. The main contributions of this work are summarized as follows. We do not regard graph convolution, attention pooling, or *k*-nearest-neighbor graphs as individually novel components; the contribution lies in their UHD-specific formulation and integration.

1. We formulate UHD-BIQA as graph-level regression over aspect-ratio-aligned native-resolution patches, which preserves local high-frequency distortion evidence while maintaining image-level prediction.
2. We construct a hybrid spatial-feature *k*-nearest-neighbor (KNN) graph and combine residual graph convolutional network (GCN) propagation with a gated attention readout for modeling global–local relations among UHD patches.
3. We adopt the exponential moving average (EMA) for multi-objective optimization and provide systematic ablations on the configuration settings under the UHD-IQA benchmark, where GLUQ achieves the lowest RMSE among the compared methods with competitive PLCC and SRCC values.

The remainder of this paper is organized as follows. Section 2 presents related work, including the definition of the UHD-BIQA problem, GRL preliminaries, GRL-based and UHD-oriented BIQA methods. Section 3 describes the technical details from aspect-ratio-aligned patching, hybrid KNN graph construction, residual GCN, gated attention, a hybrid loss function, computational complexity and implementation details. The UHD-IQA benchmark database, involved BIQA methods, evaluation metrics, performance results, ablation studies and complexity analysis are presented in Section 4. After that, the results and limitations are discussed in Section 5, and the study is concluded in Section 6.

## 2. Related Work

### 2.1. The Definition of the UHD-BIQA Problem

Given an input image, $I\in R^{H\times W\times C}$ (C=3), and its corresponding score, y∈R, obtained from human subjective studies, BIQA aims to learn a regression function $f_{\theta }$ such that $\hat{y}=f_{\theta }\left(I\right)$ approximates *y*. In the UHD settings, feeding a full-resolution image into a deep learning model is often infeasible due to memory constraints. A common workaround is to use a set of patches ${\left\{x_{i}\right\}}_{i=1}^{N}$ sampled from I and predict the image-level quality by aggregating patch-level evidence as

(1) $$\hat{y}=g_{\theta }\;\left({\left\{\phi_{\theta }\left(x_{i}\right)\right\}}_{i=1}^{N}\right)$$

where $\phi_{\theta }$ is a patch encoder, and $g_{\theta }$ is a function to predict the scores of input images.

Since the image resolution is extremely high and localized artifacts occupy only a small portion of the image, an effective UHD-BIQA method should, on the one hand, provide sufficiently dense spatial coverage to capture such localized degradations, and on the other hand, preserve global structural context to interpret these local artifacts within a coherent scene-level understanding.

### 2.2. GRL Preliminaries

GRL learns embeddings that jointly encode node attributes and graph topology. GCNs extend convolutional operations to graph-structured data via neighborhood message passing [27,28,29,30,31,32,33,34]. In the BIQA task, graph formulation has been explored from distortion graph modeling [27], non-local dependency modeling [28], adaptive graph attention [29] and inter-patch message passing [30]. Recent efforts enrich graph-based BIQA with more flexible relation modeling [28] and feature fusion [29]. Attention-based propagation [31] and expressive graph isomorphism networks [32] have also been used in computer vision, suggesting that richer message passing may benefit the BIQA task.

GRL models relational data as G=(V,E), where V is the node set and E is the edge set [33]. Each patch corresponds to a node with a feature vector, and edges encode neighborhood relations. A typical message passing layer updates node embeddings by aggregating information from neighbors. The GCN [34] computes as

(2) $$H^{\left(l+1\right)}=\sigma \;\left({\tilde{D}}^{-\frac{1}{2}}\tilde{A}{\tilde{D}}^{-\frac{1}{2}}H^{\left(l\right)}W^{\left(l\right)}\right)$$

where $\tilde{A}=A+I$ adds self-loops, $\tilde{D}$ is the degree matrix of $\tilde{A}$, $W^{\left(l\right)}$ is learnable, and σ(·) is a nonlinearity. Many inductive variants exist, such as graph attention networks [31], which differ in aggregation functions and attention mechanisms. For graph-level prediction, node representations are aggregated by a readout function for scoring image-level quality.

### 2.3. GRL-Based BIQA Methods

Several GRL-based BIQA methods have been designed from the perspectives of distortion graph representation, semantic dependency reasoning and region-level interaction modeling. Sun et al. focus on distortion-centered modeling which learns distortion graph representation over distortion types and distortion levels, and this kind of explicit relation modeling improves BIQA performance beyond isolated feature vectors [27]. Jia et al. model non-local dependencies by constructing graph nodes from superpixels and learning long-range regional interactions, which provides a complementary graph construction strategy to regular patch-based modeling [28]. Wang et al. strengthen the region-level interaction modeling via adaptive graph attention that enhance both local information and contextual interactions by refining post-Transformer features [29]. Liu et al. construct patch graphs and enable inter-patch message passing, and explicit information exchange across image regions is found to be beneficial for the BIQA task [30]. Xu et al. construct a spatial view-port graph and performing GCN reasoning across selected view-ports for omnidirectional image quality prediction [35]. Xie et al. introduce an image quality knowledge graph which is aligned with explanatory text descriptions by exploring multi-modal input, and graph structures can also support more interpretable and knowledge-driven quality prediction [36]. These studies broaden the scope of GRL-based BIQA beyond patch regression and demonstrate the flexibility of relational modeling for perceptual quality prediction.

### 2.4. UHD-Oriented BIQA Methods

UHD-oriented BIQA methods have attracted increasing attention due to the rapid growth of high-resolution visual content. Sun et al. assess UHD image quality from aesthetics, distortions, and saliency by evaluating resized images, grid-sampled local regions, and saliency-guided patches [24]. In the Challenge, grid mini-patch sampling and pyramid perception are explored to strengthen local evidence modeling under a stronger Transformer backbone [25]. Gu et al. consider pseudo-reference modeling, where original patches and super-resolution-reconstructed pseudo references are paired to learn comparative quality cues [26]. These methods show that UHD-BIQA requires more careful sampling and feature fusion. However, most existing UHD-oriented methods still rely primarily on multi-view sampling, cropping, or multi-branch fusion, while explicit patch-to-patch graph reasoning remains less explored [25].

In summary, existing UHD-specific BIQA methods preserve global or local cues through resizing, multi-view sampling, or late fusion, but they often process the sampled regions independently and lack explicit patch-to-patch relation modeling. Existing graph-based BIQA methods capture relational structure, yet they are mainly designed for standard-resolution distortions, distortion graphs, or superpixel/non-local regions rather than aspect-ratio-aligned UHD patch graphs. This gap motivates the proposed patch-graph formulation, which combines aspect-ratio-aligned UHD patch sampling with hybrid spatial-feature graph reasoning for global–local quality representation.

More broadly, the proposed global-local graph aggregation is related in spirit to graph basis function partition-of-unity methods (GBF-PUM) [37,38], which organize graph-localized basis functions through graph partitioning or community detection and reconstruct global graph signals via partition-of-unity weights. GBF-PUM targets analytical graph signal approximation, whereas GLUQ learns patch embeddings and edge affinities end-to-end for data-driven quality prediction. The shared local-to-global perspective suggests that community-aware graph pooling could be a promising direction for improving the efficiency and interpretability of UHD-BIQA.

## 3. The Proposed GLUQ Framework

**Figure 1:**
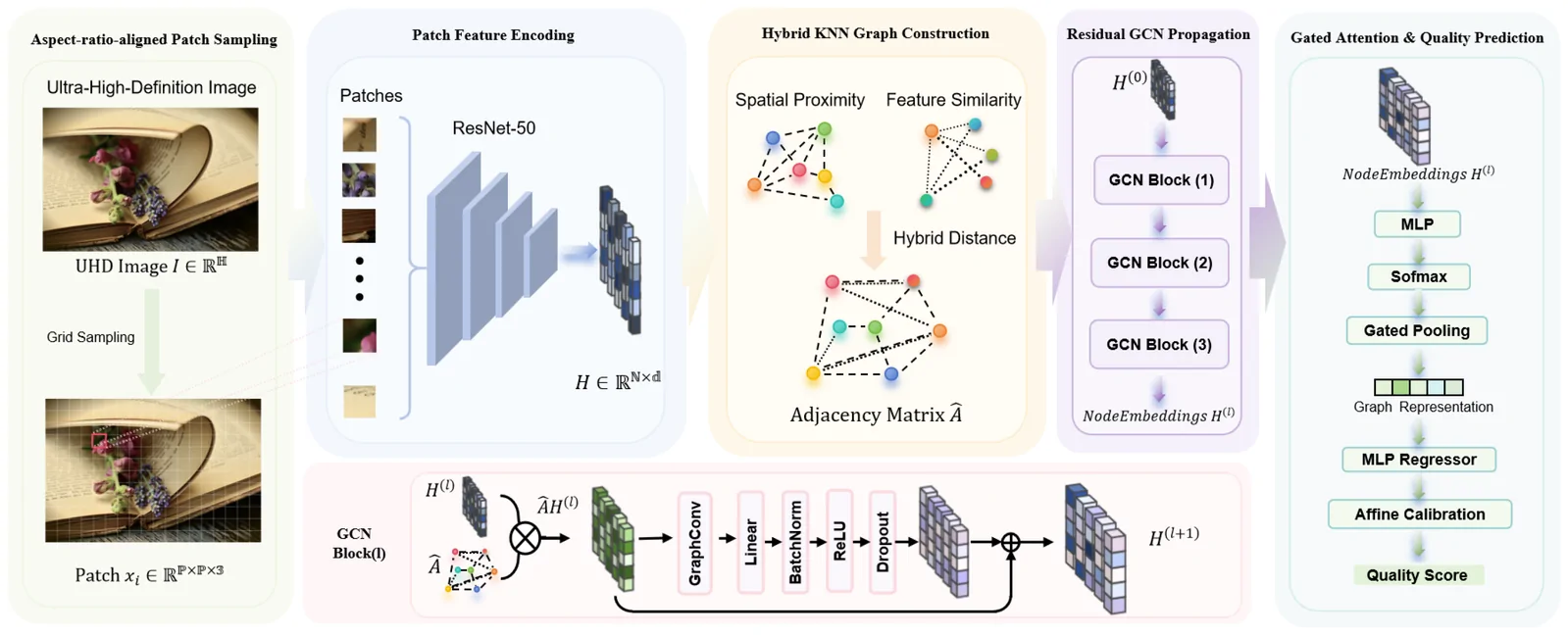
The GLUQ framework. A UHD image is partitioned into a number of aspect-ratio–aligned patches from which local features are extracted by using a pre-trained network. Then, a hybrid KNN graph is built to connect adjacent patch regions, and residual GCN layers are used to propagate contextual information. In the end, gated attention pooling aggregates the patch-level representations to produce an image-level quality prediction.

Figure 1 illustrates the proposed framework GLUQ. It consists of five steps, including aspect-ratio-aligned patch sampling, patch feature encoding, hybrid KNN graph building, residual GCN propagation, and gated attention readout for image-level score prediction.

### 3.1. Aspect-Ratio-Aligned Patch Sampling

Given an image I with width *W* and height *H*, $N=G_{w}G_{h}$ patches of size P×P are sampled on a proper rectangular grid $\left(G_{w},G_{h}\right)$. Let $\left(x_{i},y_{i}\right)$ denote the center of the *i*-th patch in pixel coordinates, and the patch position of $p_{i}$ is normalized as in Equation (3).

(3) $$p_{i}=\left[\frac{x_{i}}{W},\frac{y_{i}}{H}\right]\in {\left[0,1\right]}^{2}$$

Then, each patch is encoded by an offline backbone (ResNet-50 [39] pre-trained on ImageNet [40]) into a feature vector $h_{i}\in R^{d}$. Rectangular grids (e.g., 18×12) align better with common UHD aspect ratios (e.g., 16:9) and yield more uniform spatial coverage under a fixed patch budget.

After patch sampling, each patch is resized to the backbone input resolution, and the global average pooled feature of dimension d=2048 is extracted, followed by a linear projection to d=512 for node embedding.

The key idea of aspect-ratio-aligned patch sampling is to match the sampling lattice to the native aspect ratio rather than squeezing the entire image into a square tensor. Given a patch budget *N*, we choose a rectangular grid $\left(G_{w},G_{h}\right)$ such that $G_{w}/G_{h}\approx W/H$, so that the spatial support of sampled patches is approximately isotropic in the *imaging coordinate system*. And therefore, a practical heuristic method could be

(4) $$G_{w}=\mathrm{round}\;\left(\sqrt{N\cdot \frac{W}{H}}\right),\;G_{h}=\mathrm{round}\;\left(\frac{N}{G_{w}}\right),$$

followed by minor adjustments to satisfy $G_{w}G_{h}=N$. The advantages of aspect-ratio-aligned patch sampling are twofold. Compared with *independent patching*, the grid ensures deterministic and uniform coverage of the full field of view. Compared with *simple resizing*, it preserves local frequency characteristics inside each patch and provides a tractable number of nodes for subsequent graph reasoning.

### 3.2. Hybrid KNN Graph Construction

**Figure 2:**
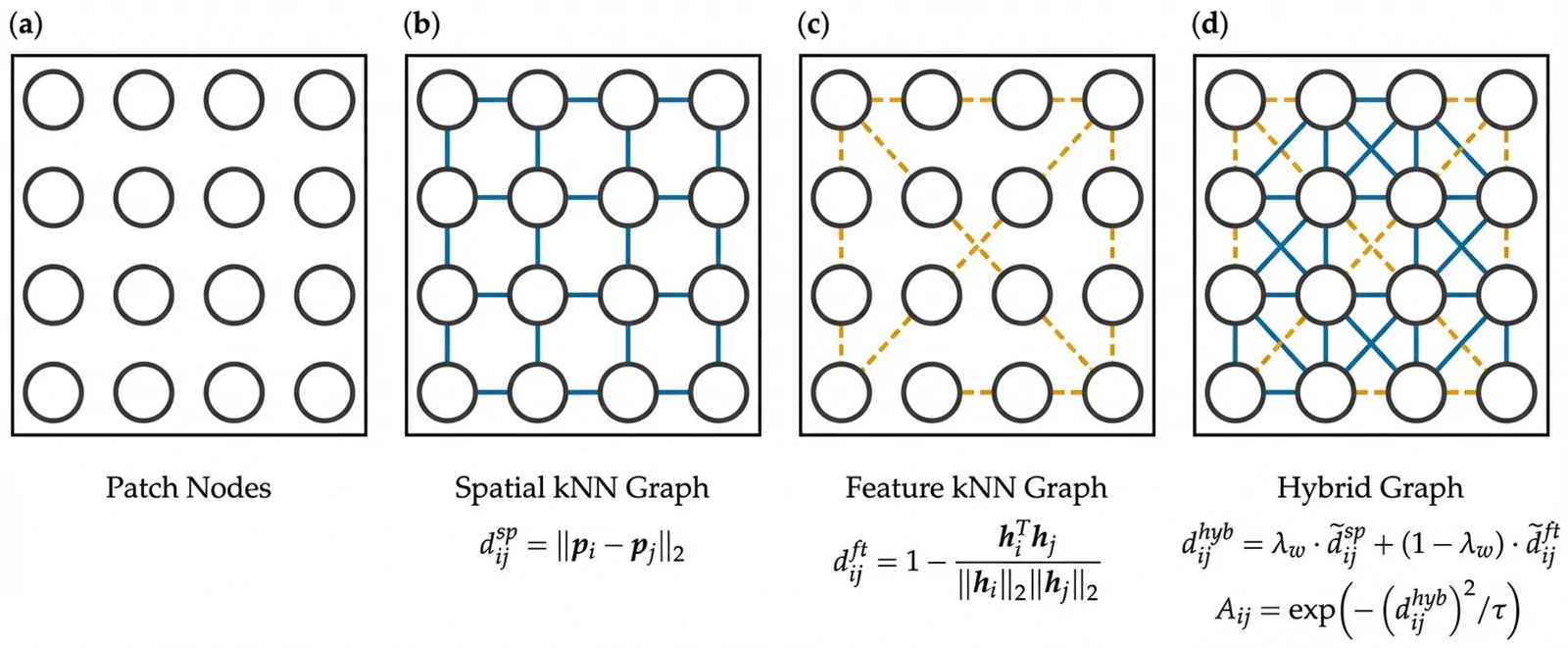
Hybrid KNN graph construction. (**a**) Patch nodes sampled on an aspect-ratio-aligned grid; each patch is treated as a node. (**b**) Spatial distance-weighted KNN graph. (**c**) Feature similarity-weighted KNN graph. (**d**) Hybrid graph that combines spatial distance and feature similarity for weighting. Edges are formed based on spatial proximity and feature similarity, and the two adjacency matrices are linearly combined to obtain the final hybrid graph used for message passing in this study.

Jointly considering spatial proximity and feature similarity, a hybrid KNN graph is built as shown in Figure 2. Based on patch nodes Figure 2a, three kinds of weighting strategies could be conducted, respectively, to form a spatial distance-weighted KNN graph Figure 2b, a feature similarity-weighted KNN graph Figure 2c, and a hybrid graph that explores both spatial distance and feature similarity for weighting Figure 2d.

For the hybrid KNN graph, Euclidean distance is defined as $d_{ij}^{sp}=\left|\right|p_{i}-p_{j}{\left|\right|}_{2}$, and feature similarity is measured as shown in Equation (5).

(5) $$d_{ij}^{ft}=1-\frac{h_{i}^{T}h_{j}}{\left|\right|h_{i}{\left|\right|}_{2}\left|\right|h_{j}{\left|\right|}_{2}}$$

Notably, the similarities $\left\{d_{ij}^{sp}\right\}$ and $\left\{d_{ij}^{ft}\right\}$ are z-score normalized to generate $\left\{{\tilde{d}}_{ij}^{sp}\right\}$ and $\left\{{\tilde{d}}_{ij}^{ft}\right\}$. And thereby, the hybrid distance metric is defined as

(6) $$d_{ij}^{hyb}=\lambda_{w}{\tilde{d}}_{ij}^{sp}+\left(1-\lambda_{w}\right){\tilde{d}}_{ij}^{ft}$$

where $\lambda_{w}\to 1$ assigns greater weight to spatial proximity.

After that, edges E={(i,j)∣j∈kNN(i)} are formed based on $d_{ij}^{hyb}$. The adjacency matrix weight $A_{ij}$ is computed as

(7) $$A_{ij}=\exp\left(-\frac{{\left(d_{ij}^{hyb}\right)}^{2}}{\tau }\right)$$

where τ is a scale parameter controlling the bandwidth of Gaussian affinity, which determines how rapidly the edge weights decay with respect to the hybrid distance [41]. In practice, τ=0.35 provides a reasonable trade-off between preserving local neighborhood structures and global connectivity.

A spatial graph preserves geometric continuity but may overlook semantically similar regions far apart, while a feature-based graph may connect such regions but ignore the strong locality prior of visual degradation. The hybrid distance metric interpolates between these two cues as shown in Equation (6), where $\lambda_{w}$ controls how strongly the graph respects geometric adjacency, and the feature term provides *content-aware shortcuts* for contextual reasoning. Notably, $\left\{d_{ij}^{sp}\right\}$ and $\left\{d_{ij}^{ft}\right\}$ are z-score normalized before mixing to prevent trivial domination by one component due to scale mismatch. Finally, the radial basis function weighting is used to transform distances into soft affinities as shown in Equation (7), which stabilizes message passing under noisy neighbor assignments.

### 3.3. Residual GCN

Given the node features $H^{\left(l\right)}\in R^{N\times d}$ at layer *l*, the graph convolution is applied as shown in Equation (8).

(8) $$U^{\left(l\right)}=\sigma \;\left(\hat{A}H^{\left(l\right)}W^{\left(l\right)}\right)$$

When *l* increases, repeated neighborhood averaging may cause node representations over-smoothing. To mitigate the over-smoothing for stable model training, residual mixing is then adopted

(9) $$H^{\left(l+1\right)}=\alpha \;U^{\left(l\right)}+\left(1-\alpha \right)\;R^{\left(l\right)}\left(H^{\left(l\right)}\right)$$

where α∈(0,1) controls aggregation strength. A smaller α preserves the original patch evidence, a larger α strengthens contextual integration, and α=0.55 is set to balance these effects.

In this study, each GCN block is implemented as a pre-activation residual unit consisting of graph convolution, linear projection, batch normalization, ReLU, and dropout as shown in Figure 1. Batch normalization stabilizes optimization under heterogeneous distortion distributions, and dropout acts as regularization against overfitting.

### 3.4. Gated Attention for Score Prediction

Node features are aggregated with a gated attention mechanism to form a graph-level representation z as shown in Equation (12). This design is related to attention-based multiple-instance learning pooling [42], which is a fit for BIQA because an image can be viewed as a “bag” of patches where only a subset may be strongly degraded. Concretely, we compute a scalar importance score for each node as

(10) $$w_{i}=g\left(h_{i}\right)$$

where g(·) is a lightweight multi-layer perceptron (MLP). The attention weights are obtained by a softmax normalization as shown in Equation (11),

(11) $$a_{i}=\frac{\exp(w_{i})}{\sum_{j}\exp\left(w_{j}\right)}$$

and the pooled representation is as shown in Equation (12).

(12) $$z=\sum_{i}a_{i}h_{i}$$

Finally, an MLP regressor f(·) predicts the raw quality score $\tilde{y}=f\left(z\right)$, and we apply an affine calibration

(13) $$\hat{y}=s\tilde{y}+b$$

to better match the score scale.

Attention pooling is important for UHD distortions because degradations are often spatially sparse. Therefore, the model must emphasize informative regions, such as blocking around edges and ringing near high-contrast structures, while it still needs to integrate global context from different patches.

### 3.5. A Hybrid Loss Function with EMA-Based Normalization

A BIQA model is to produce predictions that are accurate in absolute score with lower error, consistent in linear correlation and reliable in score ranking. To this end, we propose a composite objective function

(14) $$L\left(\theta \right)=\sum_{k\in K}\lambda_{k}\;L_{k}\left(\theta \right)$$

where θ denotes model parameters, K={mse,corr,rank,var}, and $\lambda_{k}$ are user-specified coefficients. In this study, $L_{\mathrm{mse}}$ encourages accurate regression, $L_{\mathrm{corr}}$ penalizes correlation inconsistency (e.g., 1−PLCC), $L_{\mathrm{rank}}$ enforces pairwise ordering constraints, and $L_{\mathrm{var}}$ regularizes prediction dispersion to avoid degenerate outputs.

A known issue in multi-objective training is that different objectives may have different numeric scales and gradient magnitudes. The parameter update is driven by

(15) $$\nabla_{\theta }L=\sum_{k}\lambda_{k}\;\nabla_{\theta }L_{k}$$

and if some $L_{k}$ is larger, it dominates the optimization procedure. In the hybrid loss function (Equation (15)), ranking- or correlation-based losses have bounded ranges, while the MSE loss may vary across samples in the training stage.

To mitigate this issue, we normalize each term using a running estimate of its typical magnitude. Here let $\mu_{k}\approx E\left[L_{k}\right]$ denote the characteristic scale of the *k*-th loss, and we estimate $\mu_{k}$ online with an EMA as

(16) $${\hat{\mu }}_{k}^{\left(t\right)}=\beta \;{\hat{\mu }}_{k}^{\left(t-1\right)}+\left(1-\beta \right)\;L_{k}^{\left(t\right)},\;\beta \in \left(0,1\right)$$

where *t* is the iteration index. In this study, the uncorrected EMA is sufficient when β is close to 1 and the training runs for enough iterations. Here the EMA-normalized objective is defined as

(17) $$L_{\mathrm{total}}\left(\theta \right)=\sum_{k\in K}\lambda_{k}\;\frac{L_{k}\left(\theta \right)}{\mathrm{stopgrad}({\hat{\mu }}_{k})+\epsilon }$$

where ϵ is a small constant for numerical stability, and stopgrad(·) indicates that the EMA statistics are treated as constants with respect to θ and detached from back-propagation.

And thus, the gradient of Equation (17) becomes

(18) $$\nabla_{\theta }L_{\mathrm{total}}=\sum_{k\in K}\frac{\lambda_{k}}{{\hat{\mu }}_{k}+\epsilon }\;\nabla_{\theta }L_{k}$$

which can be viewed as adaptive preconditioning that re-scales each term by the inverse of its running magnitude. Specifically, if $L_{k}$ is large, its scale estimate ${\hat{\mu }}_{k}$ grows and its effective weight is reduced. If $L_{k}$ is small, the normalization can alleviate gradient vanishing. This strategy is lightweight, suitable for the UHD-BIQA task, since patch diversity and distortion heterogeneity may cause large fluctuations across different loss terms.

### 3.6. Computational Complexity

Let $N=G_{w}G_{h}$ denote the number of patches (graph nodes), *k* the neighborhood size in the KNN graph, and *d* the node embedding dimension. Assuming a sparse adjacency with |E|≈Nk edges, one GCN layer roughly costs

(19) $$O\left(|E|d\right)+O\left(Nd^{2}\right)\approx O\left(Nkd+Nd^{2}\right)$$

where the first term corresponds to message passing and the second term corresponds to the feature transformation by $W\in R^{d\times d}$. The memory footprint is mainly from storing node features O(Nd) and sparse edges O(Nk).

In the UHD-BIQA task, the overall runtime is additionally dominated by patch feature extraction, which requires *N* forward passes through the pre-trained backbone. Therefore, *N* is a key trade-off parameter, and a larger *N* provides denser evidence while increasing both backbone and graph computing cost.

### 3.7. Implementation Details

Table 1 lists the default settings of the proposed GLUQ framework. Unless otherwise specified, all experiments and ablation studies follow the official train/validation split of the UHD-IQA benchmark database for hyper-parameter selection as well as the held-out test split for prediction performance comparison.

The GLUQ framework is implemented in PyTorch (version 2.1), and sparse graph operations are used to scale message passing to hundreds of nodes per image. To avoid data leakage, all hyper-parameters, including grid size, neighborhood *k*, distance mixing, and loss weights, are selected on the training/validation split, and the held-out test split is used for the final reported evaluation. Patch extraction uses an aspect-ratio-aligned grid, each 256×256 crop is clipped so that it stays within the image, and no padding beyond the image boundary is introduced. Patches are converted to tensors and normalized with the ImageNet suggested mean and standard deviation [40]. In the training stage, a small grid jitter is applied for augmentation, whereas in the validation and testing stages, the deterministic grid is used unless test-time augmentation is explicitly enabled. A full training run completes within 16 h on a single A800 GPU under early stopping. The trained .pt checkpoint is released at https://github.com/NicoYuCN/GLUQ (accessed on 23 July 2026) for reproducibility, whereas the full private training repository is not released at this stage. A high-level summary of the inference pipeline is provided in Appendix A (Algorithm A1).

## 4. Experiments and Results

### 4.1. The Database

The UHD-IQA benchmark database [23] contains 6,073 UHD photographs with a standardized width of 3840 pixels. Each image is annotated by 20 expert ratings aggregated into a mean opinion score (MOS). The official split includes 4269 training images, 904 validation images, and 900 testing images.

### 4.2. Involved BIQA Methods

Three categories of BIQA methods are compared. The first group includes three DL-based methods (DBCNN [11], CONTRIQUE [12] and ARNIQA [13]). The second group contains the methods from the Challenge [25]. The last consists of GRL-based methods, including GraphIQA [27], NLNet-IQA [28], AGA-IQA [29], and our proposed GLUQ framework.

### 4.3. Performance Evaluation

Three metrics, Spearman rank-order correlation coefficient (SRCC), Pearson linear correlation coefficient (PLCC), and root mean square error (RMSE), are used for evaluating prediction performance. In accordance with MOS values, a higher SRCC indicates stronger monotonic agreement, a higher PLCC reflects better linear agreement, and a lower RMSE indicates smaller absolute prediction error.

### 4.4. Prediction Performance on the UHD-IQA Benchmark Database

Table 2 shows the prediction performance from three major categories of DL-based methods, the Challenge-leading UHD-specific methods and GRL-based methods on the UHD-IQA benchmark database. For each category of BIQA method, the best metric values are shown in boldface. For fair comparison, we distinguish quoted and reproduced results. Metric values of the DL-based methods and the Challenge methods are quoted from the UHD-IQA benchmark [23] and the AIM 2024 UHD challenge report [25] respectively under the official split. The GRL-based baselines, including GraphIQA, NLNet-IQA and AGA-IQA, have no published values on this benchmark under the same protocol, so we reproduce them under the official train/validation/test split with the identical PLCC/SRCC/RMSE evaluation procedure used for GLUQ. For GLUQ, all hyper-parameters are selected on the validation split (criterion: validation SRCC), the test split is used for the final single report, and the affine calibration is a trainable readout module rather than a post hoc fit on the test set.

GLUQ achieves the lowest RMSE and slightly inferior SRCC and PLCC values compared with UIQA, GS-PIQA and CIPLAB among all the methods. Firstly, the methods from the Challenge [25] confirm the value of architectures specifically optimized for UHD settings. CIPLAB achieves the highest PLCC (0.7995) and UIQA [24] obtains the best SRCC (0.8463). These methods likely benefit from specialized patch aggregation, distortion-aware modeling, or multi-cues-driven optimization. Secondly, GRL-based methods [27,28,29] obtain relatively inferior results to the methods in the Challenge [25]. Graph modeling is suitable for preserving structural relationships among image regions, while the comparison results suggest that these approaches [27,28,29] may not fully exploit UHD-specific spatial dependencies or may suffer from sub-optimal graph construction. Notably, GLUQ achieves the best performance among the GRL-based methods with highest PLCC and SRCC and the lowest RMSE value. It enhances prediction precision by better modeling patch relationships and reducing regression error. Thirdly, the performance of DL-based approaches [11,12,13] seems limited on the UHD-IQA database [23]. In other words, these methods originally designed for standard-resolution images struggle to generalize to UHD perceptual distortions. The inferior performance indicates that DL-based strategies may inadequately capture the complex multi-scale and heterogeneous distortions present in UHD content.

### 4.5. Ablation Studies

#### 4.5.1. The Sampling Grid Number

Table 3 shows the BIQA performance and training time under different grid resolutions via a simple setting ($\lambda_{\mathrm{mse}}=1$). When the grid number increases from N=150 to N=216, both SRCC and PLCC improve noticeably, while the RMSE decreases from 0.0632 to 0.0602. This trend suggests that denser spatial sampling provides richer local evidence and helps the graph model capture more informative distortion patterns in UHD images. However, increasing grid density also leads to higher computational cost, as reflected by the longer wall-clock training time.

The 18×12 grid (N=216) offers a favorable trade-off between BIQA performance and computational feasibility. When the grid resolution increases to 21×14 (N=294), the model training becomes infeasible due to out-of-memory errors. Based on the observation, we adopt the 18×12 grid as a balanced configuration for the subsequent experiments, including the search of the graph neighborhood size and the later coefficient optimization of the full multi-loss objective.

#### 4.5.2. The Neighborhood Size *k* for KNN Graph Construction

**Figure 3:**
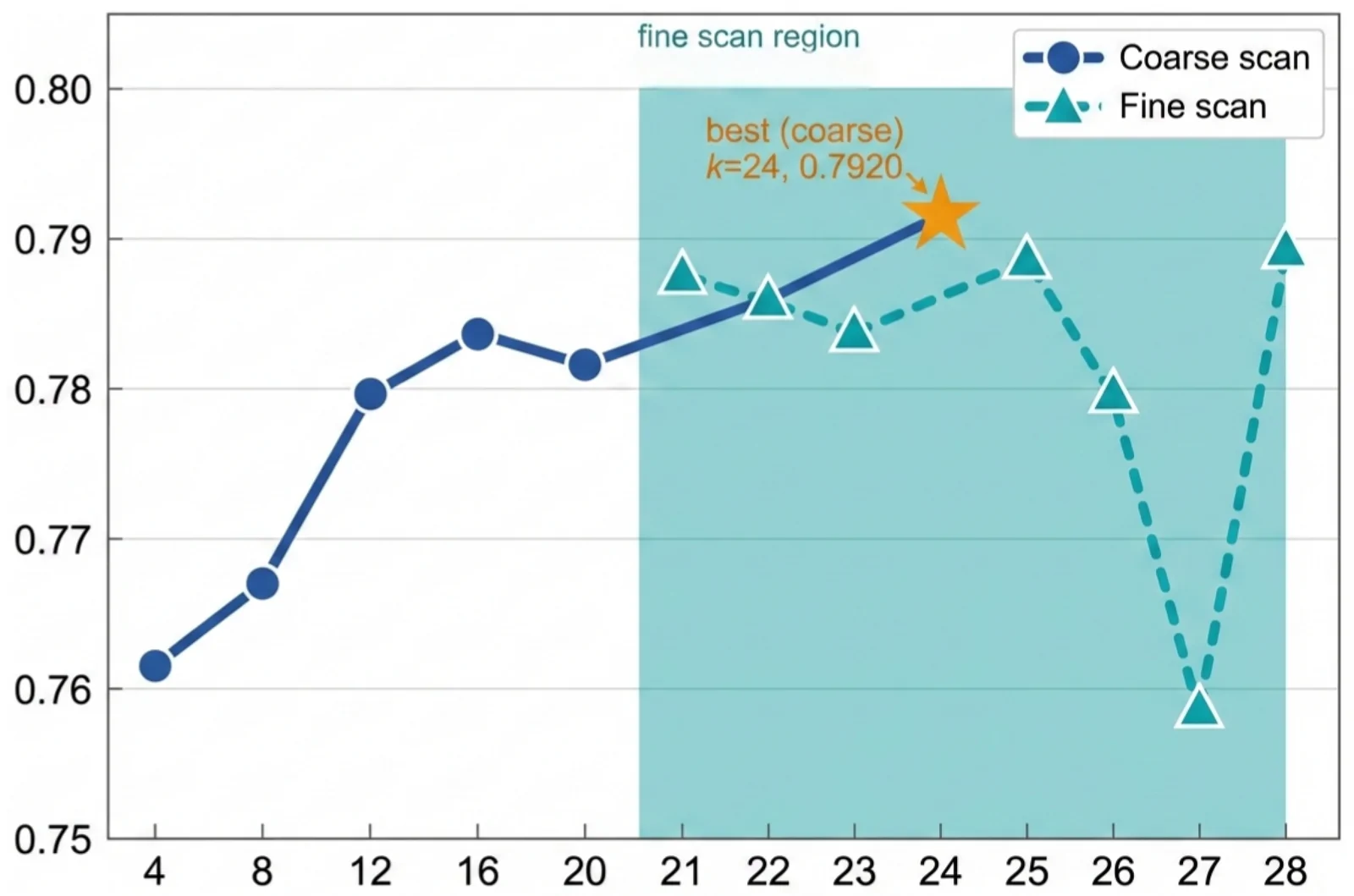
Coarse-to-fine search of the neighborhood size *k* for graph construction.

Figure 3 presents a coarse-to-fine search procedure for the neighborhood size *k* under a simple setting ($\lambda_{\mathrm{mse}}=1$). The coarse scan first evaluates a compact candidate set between 4 and 28 with an equal interval of 4, and k=24 is identified as the most promising option. A subsequent fine-grained examination around this region between 21 and 28 with an equal interval of 1 further confirms that k=24 remains the best-performing neighborhood size for graph construction.

The result indicates that a moderately sized local neighborhood is sufficient to capture the structural dependency among sampled regions, since using too few neighbors may lead to incomplete local connectivity, whereas overly large neighborhoods may introduce redundant or less informative relations.

#### 4.5.3. Coefficient Search for the Hybrid Loss Function

Under the settings of grid =18×12 and k=24, the coefficients in Equation (17) is investigated under EMA-normalized multi-objective optimization. Table 4 summarizes all tested coefficient settings, including the single-term cases and multi-loss combinations, and sorts them by SRCC in descending order.

It is found that $\lambda_{\mathrm{corr}}=0.8$, $\lambda_{\mathrm{mse}}=0.0$, $\lambda_{\mathrm{rank}}=0.2$, and $\lambda_{\mathrm{var}}=0.0$ lead to the highest SRCC value (0.7982). Several mixed settings also outperform the single-term cases, indicating that correlation and ranking objectives can complement score regression when the coefficients are chosen appropriately. By contrast, configurations dominated by the variance term ($\lambda_{\mathrm{var}}=1.0$) lead to clear performance degradation.

The observations suggest that the EMA-normalized formulation is beneficial, while its effectiveness depends on careful coefficient selection. The best-performing coefficient setting is therefore adopted in the subsequent graph-topology study, where the graph scale and neighborhood size remain fixed so that the effect of the graph distance measure can be examined in a controlled manner. This coefficient search was conducted under the default Euclidean-only distance ($\lambda_{w}=1.0$), and the best setting ($\lambda_{\mathrm{corr}}=0.8$, $\lambda_{\mathrm{rank}}=0.2$) attains PLCC/SRCC/RMSE of 0.7744/0.7982/0.0567, which corresponds exactly to the $\lambda_{w}=1.0$ row in Table 5; we then fix these coefficients and search the spatial–feature mixing weight $\lambda_{w}$, reaching the best 0.7784/0.8019/0.0519 at $\lambda_{w}=0.7$. This staged protocol keeps each ablation single-variable and makes the search traceable, while a full joint search is prohibited by the high cost of encoding hundreds of UHD patches per image.

#### 4.5.4. Effect of Different Distance Measures in Graph Construction

The graph topology is evaluated under various distance metrics and weighting schemes (Equation (6)). As shown in Table 5, we compare feature-based, Euclidean-based, and hybrid KNN graph constructions. The results consistently demonstrate that spatial proximity is the primary factor governing the effectiveness of graph representation.

As shown in Table 5, incorporating spatial distance into graph construction significantly improves performance over the feature-only baseline. The hybrid graph exhibits a consistent performance gain as $\lambda_{w}$ increases from 0.1 to 0.7, indicating that spatial information effectively regularizes graph connectivity and enhances perceptual consistency. The best performance is achieved at $\lambda_{w}=0.7$, suggesting an optimal balance between spatial proximity and feature similarity. Further increasing $\lambda_{w}$ leads to performance degradation, implying that excessive reliance on spatial structure suppresses discriminative feature relationships. Notably, the spatial-only graph ($\lambda_{w}=1.0$) remains competitive but does not surpass the optimal hybrid setting, demonstrating that spatial information is more important for graph construction.

### 4.6. Computing Complexity

Table 6 compares the computational complexity of three UHD-BIQA frameworks in terms of input size, parameter count, and multiply–accumulate operations (MACs). GLUQ has the smallest model size, indicating strong parameter efficiency, but it also requires substantially higher MACs.

GLUQ offers the most parameter-efficient design among the approaches, requiring only 25.61 M parameters compared with 82.85 M for UIQA and 144.81 M for GS-PIQA. This lightweight model size reduces storage demands and simplifies deployment. However, these advantages come at the cost of substantially higher computational complexity, with GLUQ requiring 1153.59 GMACs, far exceeding the 43.53 GMACs and 50.26 GMACs required by the compared methods. This heavy computational burden is mainly caused by its multi-patch encoding and graph reasoning strategy, which enhances detailed perceptual modeling for UHD images while significantly increases inference cost. Overall, GLUQ achieves a favorable balance in model compactness and training simplicity, while its high MACs indicate further optimization for real-time or resource-constrained applications.

### 4.7. Further Analysis and Stability

Beyond the single-configuration comparison, we further examine the parameter stability, statistical reliability, computational behavior, and backbone capacity of GLUQ under the default configuration (grid 18×12, k=24, $\lambda_{w}=0.7$, τ=0.35, α=0.55, dropout 0.15, AdamW with initial learning rate $2\times 10^{-4}$, and the EMA-normalized loss with $\lambda_{\mathrm{corr}}=0.8$ and $\lambda_{\mathrm{rank}}=0.2$).

#### 4.7.1. Parameter Interaction and Local Stability

The hybrid graph depends on several interacting design choices. In view of the high cost of encoding hundreds of UHD patches per image, these factors are examined in a staged manner rather than by an exhaustive joint search (Section 4.5.3). To verify that the selected configuration is not a fragile single-point optimum, we report local scans around it under the default multi-objective setting.

Table 7 shows local stability when the neighborhood size *k* changes where τ=0.35. It is found that the neighborhood size k∈{22,…,26} yields an SRCC variation below 0.002 with the peak at k=24 that leads to stable UHD image quality prediction.

Table 8 illustrates the local stability when the Gaussian bandwidth τ changes. It is observed that τ∈[0.25,0.50] similarly varies by less than 0.003 with the peak at τ=0.35. In summary, the selected configuration of *k* and τ lies within a region for stable UHD-BIQA performance.

#### 4.7.2. Multi-Seed Stability and Confidence Intervals

To assess the robustness of model initialization and data ordering, the default configuration is used with three random seeds (Table 9). Notably, the reported result corresponds to the validation-selected checkpoint of seed 1337. As shown in Table 9, small standard deviations (PLCC 0.7781±0.0033, SRCC 0.8016±0.0026, RMSE 0.0520±0.0005) are observed, indicating low sensitivity of UHD-BIQA prediction performance to random seeds.

We additionally report bootstrap 95% confidence intervals (CIs) over 10,000 resamples of the test images, since the test set is of moderate size. As shown in Table 10, the RMSE interval upper bound (0.0548) does not overlap with the strongest competitor GS-PIQA (0.0607), supporting the “lowest RMSE” claim, whereas the PLCC/SRCC intervals overlap with the top Challenge methods.

#### 4.7.3. Effect of EMA Normalization Versus Auxiliary Objectives

To disentangle the contribution of EMA normalization from that of adding correlation/ranking objectives, Table 11 compares six controlled settings. Adding the correlation/ranking objectives improves SRCC by 0.0032 and RMSE by 0.0040 over MSE-only, and EMA normalization further improves SRCC by 0.0045 and RMSE by 0.0019 under the same multi-objective weights while stabilizing training, whereas EMA alone on a single MSE loss is nearly inert. EMA normalization thus acts as a scale normalizer for heterogeneous losses rather than a universal stabilizer.

#### 4.7.4. Inference Profiling and Deployment Cost

Table 12 reports the per-image inference cost on a single A800 80 GB GPU (batch size 1, AMP, no TTA). It is found that the runtime is dominated by repeated patch-level ResNet-50 extraction (≈72% of the 323 ms per-image latency), whereas graph construction and the three-layer GCN together account for only ≈12%. Peak memory and throughput scale from 5.8 GB and 3.1 img/s at batch size 1 to 32.4 GB and 12.7 img/s at batch size 8, and enabling TTA (two-pass plus horizontal flip) raises the per-image latency to 690 ms.

Therefore, GLUQ is much suitable for offline or near-offline UHD image quality prediction than for real-time monitoring, while acceleration should prioritize fewer patches, shared backbone feature maps, lighter backbones, or community-aware graph pooling.

#### 4.7.5. Backbone Capacity

To test whether a stronger backbone improves correlation, we replace ResNet-50 [39] with Swin-T [43] while keeping the graph, GCN, readout, and loss fixed, projecting the final-stage Swin-T feature to the same 512-*d* node embedding.

Table 13 shows that Swin-T improves PLCC/SRCC by about 0.011 and slightly lowers RMSE, while it increases parameters by 20%, MACs by 45%, per-image latency from 323 ms to 412 ms, and peak memory from 45–58 GB to 55–68 GB. ResNet-50 is therefore retained as the efficiency-balanced default, and the result indicates that backbone capacity is one factor that may partly limit the correlation performance of GLUQ rather than the sole cause of the gap to the strongest Challenge methods.

### 4.8. Qualitative Behavior of the Attention Readout

**Figure 4:**
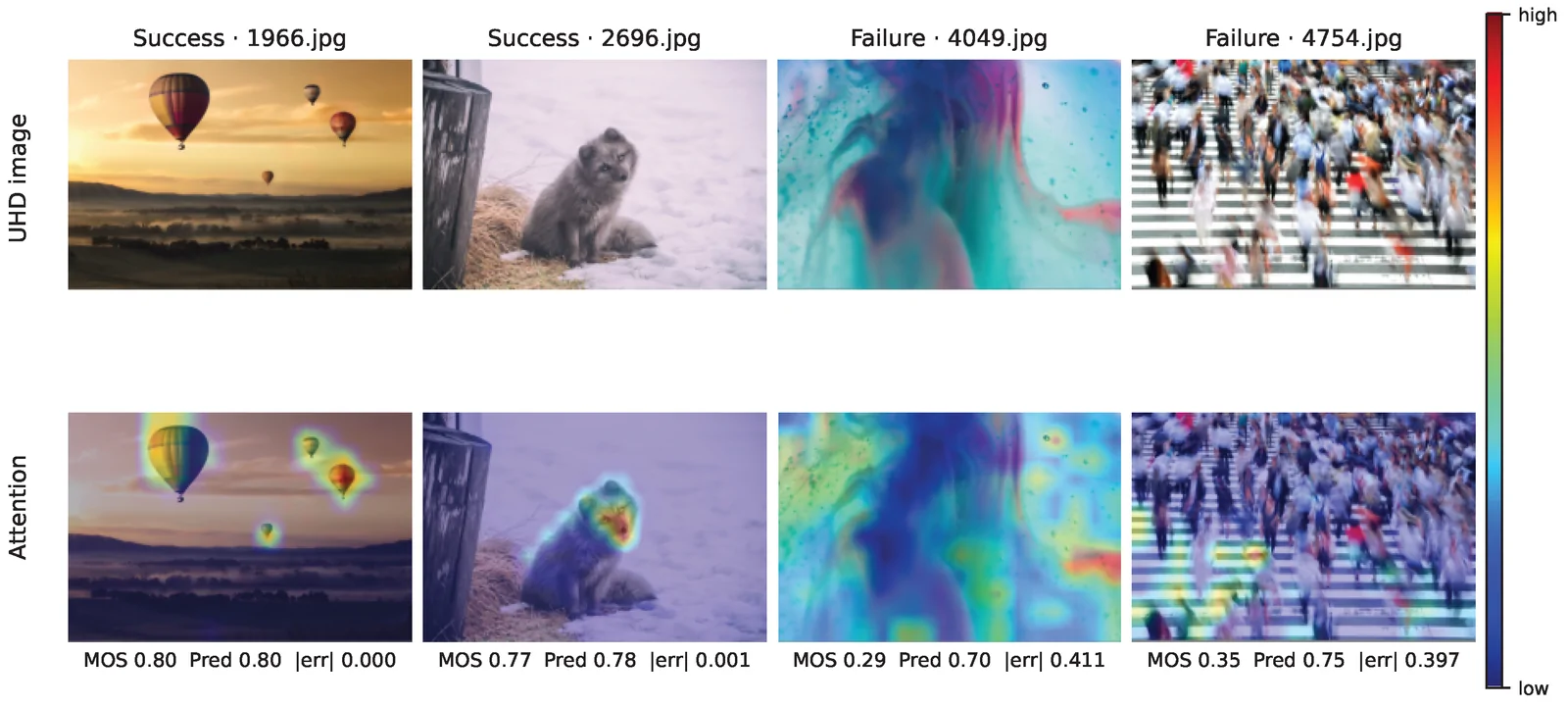
Gated-attention behavior of the trained GLUQ model. The 216 patch-level attention weights are reshaped to the 12×18 sampling grid, bilinearly upsampled, and overlaid (jet colormap; red = high weight, per-image min–max normalized) on a downsampled copy of each UHD image. MOS, the predicted score, and the absolute error are reported under each overlay. Left two columns are success predictions. Attention concentrates on perceptual regions of interest—balloon contours (1966) and the subject’s face (2696). Right two columns are failure cases. In 4049, it is dispersed over colorful droplets while the severely blurred center is underweighted, while in 4754, the readout favors crisp, repetitive crosswalk stripes over the pervasive motion-blurred pedestrians.

To examine whether the learned gated readout attends to quality-relevant regions, Figure 4 visualizes the 216 patch-level attention weights of a trained GLUQ model, reshaped to the 12×18 sampling grid, bilinearly upsampled, and overlaid on the image. Since the map has only 12×18 support, it indicates which patch groups dominate the readout, not pixel-precise localization. In successful predictions, the weights concentrate on perceptual regions of interest (e.g., the subject’s face and salient object contours), whereas in two failure cases the readout is misled. The attention readout favors crisp repetitive high-frequency texture (crosswalk stripes) over the genuinely degraded regions (motion-blurred pedestrians), or becomes dispersed over colorful but uninformative structure. This indicates that gated attention offers useful content-aware quality sampling for spatially sparse UHD distortions, yet is not strictly distortion-specific and can be biased by salient texture.

## 5. Discussion

UHD-BIQA is challenging due to the extremely high spatial resolution of the input images. To address this challenge, GLUQ is proposed (Figure 1) that combines aspect ratio-aligned patch sampling, hybrid KNN graph construction, and EMA-based optimization of a hybrid loss function. The experimental results suggest that GLUQ achieves promising performance on the UHD-IQA database (Table 2) based on the grid patch number N=18×12 (Table 3), k=24 in KNN graph building (Figure 3), $\lambda_{corr}=0.8$ and $\lambda_{rank}=0.2$ in the hybrid loss function (Equation (14)), and $\lambda_{w}=0.7$ in the weighted distance metric (Equation (6)).

### 5.1. Why Graph Reasoning Is Suitable for the UHD-BIQA Task

A practical advantage of graph reasoning for UHD-BIQA is that it places all sampled patches into a unified relational structure instead of treating them as isolated crops. This matters because UHD quality is rarely determined by a single local artifact alone; rather, local degradations are interpreted in the context of surrounding regions and the broader scene layout. *Simple resizing* and *independent patching* preserve some local detail, while they usually fuse different views after separate feature extraction [24,25,26]. By contrast, graph message passing provides an explicit mechanism for contextual interaction among all sampled regions, and gated readout lets the final prediction emphasize the most informative regions without discarding scene-level context [27,28,29,30].

The experimental results are consistent with this interpretation. Table 3 shows that performance improves when the sampling grid becomes dense enough to expose richer local evidence, and Figure 3 shows that graph reasoning is most effective only when the neighborhood size is chosen carefully rather than enlarged arbitrarily. Table 2 further shows that the resulting pipeline achieves the best RMSE among the public UHD-oriented methods. Taken together, these observations support the basic motivation behind graph reasoning for UHD-BIQA that local evidence is useful, and it becomes more reliable when it is embedded in a structured global context rather than pooled independently. This is also consistent with scale-aware UHD studies [21,22], which suggest that perceptual quality is entangled with native spatial structure.

### 5.2. Why GLUQ Does Not Outperform the Top-Tier Methods in the Challenge

GLUQ does not outperform the top-tier methods in the Challenge [24,25], and reasons are manifold. Generally, UIQA [24] combines global aesthetics, local distortion fragments, and saliency-guided regions through coordinated Transformer branches. GS-PIQA [25] enhances ranking-oriented perceptual modeling by incorporating grid mini-patch sampling, pyramid perception, and a larger Swin-base backbone under the challenge setting. These models achieve superior performance by leveraging advanced backbone architectures, enriched feature representations, and effective multi-scale semantic integration.

GLUQ exhibits slightly inferior correlation performance while achieving the lowest RMSE (Table 2). The effectiveness of GLUQ can be attributed to several factors. First, the framework unifies patch encoding, graph construction, and interactive message passing within a single structured pipeline. This design enables more accurate absolute score calibration, as reflected by its superior RMSE performance. Second, GLUQ employs a pre-trained ResNet-50 [39] as the feature extractor, which is computationally efficient but less expressive than the more advanced backbone architectures used in UIQA [24] and GS-PIQA [25], as also evidenced by the controlled backbone ablation in Table 13. Therefore, GRL-based patch reasoning facilitates effective global–local contextual interaction in UHD image quality representation, thereby improving UHD-BIQA performance.

### 5.3. Computational Considerations

One challenging issue of GLUQ lies in its high MACs (Table 6), particularly when hundreds of patches are aggregated to enhance UHD perceptual representation (Table 3). The computational cost is in fact dominated by repeated patch-level feature extraction rather than by edge-level message passing. Profiling (Table 12) shows that ResNet-50 patch encoding accounts for about 72% of the per-image latency, whereas graph construction and the GCN together account for only about 12%, so the large MAC count is driven primarily by the hundreds of native-resolution patch encodings. Moreover, when attention mechanisms and pairwise similarity modeling are introduced, additional overhead arises from dense relational computations, significantly increasing complexity in high-dimensional feature space. Consequently, improving the real-time efficiency of GLUQ becomes an important requirement for enabling practical deployment in time-sensitive UHD-BIQA scenarios.

### 5.4. Limitations of the Current Study

The current study has several limitations. One constraint of GLUQ lies in its heavy computational cost (Table 6) which hampers its applications in time-sensitive UHD-BIQA scenarios, in particular when denser patch sampling can improve prediction accuracy (Table 3). Beyond efficiency, the representational capacity of GLUQ is limited when compared with UHD-specific Transformer-based or multi-branch systems [25]. The backbone ablation (Table 13) indicates that the representation capacity of ResNet-50 [39] may partly limit the correlation performance: replacing it with Swin-T [43] improves PLCC/SRCC by about 0.011 at the cost of substantially more parameters, MACs, and latency, so the gap to the strongest Challenge methods is unlikely to be explained by the backbone alone. Meanwhile, graph construction requires further investigation. The ablation studies show that the final performance is affected by several factors, including the sampling grid, the neighborhood size *k*, the multi-loss coefficients, and the spatial-feature mixing weight $\lambda_{w}$. Owing to the high computational cost, these factors are examined in a staged manner rather than exhaustive joint optimization. The attention analysis is qualitative and the gated readout is not strictly distortion-specific. The Swin-T ablation is a single-run exploratory study. Finally, the scope of the experimental validation is limited to a single benchmark, and broader testing on additional databases is needed to examine cross-database generalization.

## 6. Conclusions

In this study, GLUQ is proposed for alleviating the challenges associated with extremely high resolution and global–local inconsistency in the UHD-BIQA task. It combines aspect ratio-aligned patching, KNN-based graph construction, and multi-objective loss weighting to predict image-level quality scores by propagating information among local representations through GCN-based message passing. On the UHD-IQA benchmark database, GLUQ obtains the lowest RMSE among the compared methods with competitive PLCC and SRCC, and the supplementary stability, confidence-interval, profiling, and attention analyses support the reliability and bounded behavior of the framework. Future work will explore more efficient patch encoding, shared backbone feature maps, adaptive patch selection, community-aware graph pooling, and stronger visual backbones to improve the real-time efficiency of UHD-BIQA, and will further validate GLUQ on additional databases to examine cross-database generalization.

## Figures and Tables

**Table 1:** Default implementation details of the proposed GLUQ.

Component	Settings
Patch sampling	Patch size P=256; grid $\left(G_{w},G_{h}\right)=\left(18,12\right)$ (N=216)
Backbone encoder	ResNet-50 [39] pre-trained on ImageNet [40]
Node embedding	Global average pooled 2048-dimensional (*d*) features are linearly projected to d=512 features
Graph construction	k=24; edge weight via radial basis function kernel (Equation (7))
Distance metric	Hybrid spatial–feature distance with $\lambda_{w}=0.7$ (Equation (6))
GCN	L=3 residual layers; the mixing coefficient α=0.55
Readout	Gated attention pooling with MLP regression; affine calibration
Regularization	Dropout 0.15; batch normalization in each GCN block
Optimization	AdamW; initial learning rate $2\times 10^{-4}$ (backbone × 0.5); weight decay $6\times 10^{-5}$; gradient clipping 1.0
Training schedule	40 epochs; 3-epoch warmup; early stopping with patience 5; batch size 6 with gradient accumulation 6 (effective 36); mixed-precision (FP16); training-time grid jitter 0.08
Model selection	Best checkpoint selected by validation SRCC; EMA of weights with decay 0.999 used for evaluation
Evaluation	EMA weights; two-pass plus horizontal-flip test-time augmentation (TTA); hyper-parameter selection
Hardware	NVIDIA A800 GPU (80 GB)
Reproducibility	Trained checkpoint (.pt) to be released for research use; full training repository not released

**Table 2:** Comparison of different categories of methods on the UHD-IQA benchmark database. Entries marked with ^†^ are reproduced under the official split with identical evaluation procedure, and the other entries are quoted from [23,25]. Bold indicates the best overall result of our proposed method (GLUQ).

Categories	Algorithm	PLCC	SRCC	RMSE
DL-based [23]	DBCNN [11]	**0.723**	**0.745**	0.154
	CONTRIQUE [12]	0.678	0.732	**0.073**
	ARNIQA [13]	0.694	0.739	0.074
The Challenge [25]	UIQA [24]	0.7985	**0.8463**	0.0615
	GS-PIQA	0.7925	0.8297	**0.0607**
	CIPLAB	**0.7995**	0.8354	0.0638
	EQCNet	0.7682	0.7954	0.0621
	MobileNet-IQA	0.7559	0.7883	0.0659
	NF-RegNets	0.7222	0.7715	0.0703
GRL-based	GraphIQA ^†^ [27]	0.6420	0.6632	0.0875
	NLNet-IQA ^†^ [28]	0.6804	0.7122	0.0675
	AGA-IQA ^†^ [29]	0.5914	0.6426	0.0972
	GLUQ (ours)	**0.7784**	**0.8019**	**0.0519**

**Table 3:** BIQA performance and time cost regarding the grid size. Arrows indicate the preferred direction (↑: higher is better; ↓: lower is better).

Grid	*N*	Best *k*	SRCC ↑	PLCC ↑	RMSE ↓	Time (min) ↓
15 × 10	150	20	0.7791	0.7281	0.0632	485.8
18 × 12	216	24	**0.7920**	**0.7545**	**0.0602**	603.0
21 × 14	294	–	–	–	–	–

**Table 4:** Coefficients via EMA-based multi-objective optimization (sorted by SRCC). Arrows indicate the preferred direction (↑: higher is better; ↓: lower is better).

$\lambda_{\mathrm{corr}}$	$\lambda_{\mathrm{mse}}$	$\lambda_{\mathrm{rank}}$	$\lambda_{\mathrm{var}}$	PLCC ↑	SRCC ↑	RMSE ↓
0.8	0.0	0.2	0.0	0.7744	**0.7982**	0.0567
0.6	0.4	0.0	0.0	0.7548	0.7939	0.0540
0.0	1.0	0.0	0.0	0.7545	0.7920	0.0602
0.4	0.6	0.0	0.0	0.7540	0.7919	0.0611
0.2	0.0	0.8	0.0	0.7626	0.7914	0.0576
0.2	0.8	0.0	0.0	0.7495	0.7906	0.0601
1.0	0.0	0.0	0.0	0.7673	0.7897	0.0612
0.0	0.8	0.2	0.0	0.7439	0.7860	0.0620
0.8	0.2	0.0	0.0	0.7464	0.7847	0.0536
0.8	0.0	0.0	0.2	0.7516	0.7828	0.0619
0.0	0.6	0.4	0.0	0.7476	0.7820	0.0540
0.0	0.4	0.6	0.0	0.7489	0.7799	0.0574
0.0	0.0	0.6	0.4	0.7621	0.7780	0.0567
0.6	0.0	0.4	0.0	0.7465	0.7730	0.0570
0.0	0.0	0.8	0.2	0.7381	0.7694	0.0584
0.4	0.0	0.6	0.0	0.7427	0.7678	0.0568
0.0	0.8	0.0	0.2	0.7160	0.7508	0.0455
0.0	0.0	1.0	0.0	0.7220	0.7426	0.0594
0.0	0.2	0.8	0.0	0.7565	0.7201	0.0599
0.0	0.0	0.4	0.6	0.6410	0.6591	0.0601
0.6	0.0	0.0	0.4	0.6158	0.6476	0.0575
0.4	0.0	0.0	0.6	0.3670	0.3955	0.0572
0.0	0.0	0.2	0.8	0.2904	0.3363	0.0573
0.0	0.4	0.0	0.6	0.1384	0.2932	0.0639
0.2	0.0	0.0	0.8	0.2283	0.2847	0.0578
0.0	0.2	0.0	0.8	0.1475	0.2614	0.0630
0.0	0.0	0.0	1.0	0.1316	0.2421	0.0662
0.0	0.6	0.0	0.4	0.0635	0.2227	0.0640

**Table 5:** Effect of graph distance measures under different weights $\lambda_{w}$. Arrows indicate the preferred direction (↑: higher is better; ↓: lower is better).

	$\lambda_{w}$	PLCC ↑	SRCC ↑	RMSE ↓
Feature-only (semantic)	0.0	0.7216	0.7434	0.0728
Hybrid	0.1	0.7466	0.7683	0.0672
Hybrid	0.3	0.7682	0.7894	0.0593
Hybrid	0.5	0.7741	0.7933	0.0598
Hybrid	0.7	**0.7784**	**0.8019**	**0.0519**
Hybrid	0.9	0.7709	0.7915	0.0617
Euclidean-only (spatial)	1.0	0.7744	0.7982	0.0567

**Table 6:** Comparison of computing complexity across current UHD-BIQA frameworks.

	Input Size	Params. (M)	MACs (G)
UIQA [24]	480×480	82.85	43.53
GS-PIQA [25]	384×384	144.81	50.26
GLUQ (ours)	(18×256)×(12×256)	25.61	1153.59

**Table 7:** Local ↑: higher is better; ↓: lower is better. Bold marks the best result. stability when the neighborhood size *k* changes. Arrows indicate the preferred direction (↑: higher is better; ↓: lower is better); bold marks the best result.

*k*	PLCC ↑	SRCC ↑	RMSE ↓
22	0.7761	0.8004	0.0522
23	0.7770	0.8010	0.0521
**24**	**0.7784**	**0.8019**	**0.0519**
25	0.7780	0.8016	0.0520
26	0.7772	0.8011	0.0520
27	0.7751	0.7996	0.0524
28	0.7736	0.7985	0.0527

**Table 8:** Local stability when the Gaussian bandwidth τ changes. Arrows indicate the preferred direction (↑: higher is better; ↓: lower is better).

τ	PLCC ↑	SRCC ↑	RMSE ↓
0.25	0.7762	0.7998	0.0524
0.30	0.7778	0.8014	0.0521
**0.35**	**0.7784**	**0.8019**	**0.0519**
0.40	0.7779	0.8015	0.0521
0.50	0.7761	0.8001	0.0525

**Table 9:** Multi-seed stability of the default configuration. Arrows indicate the preferred direction (↑: higher is better; ↓: lower is better).

Seed	PLCC ↑	SRCC ↑	RMSE ↓
1337 (reported)	0.7784	0.8019	0.0519
2024	0.7746	0.7988	0.0525
3407	0.7812	0.8040	0.0515
Mean ± std	0.7781±0.0033	0.8016±0.0026	0.0520±0.0005

**Table 10:** Bootstrap 95% CIs over 10,000 resamples of the test images. Arrows indicate the preferred direction (↑: higher is better; ↓: lower is better).

Metric	Point Estimate	95% CI
PLCC ↑	0.7784	[0.7510, 0.8048]
SRCC ↑	0.8019	[0.7776, 0.8247]
RMSE ↓	0.0519	[0.0492, 0.0548]

**Table 11:** The effect of EMA normalization from that of auxiliary correlation/ranking objectives. Arrows indicate the preferred direction (↑: higher is better; ↓: lower is better); bold marks the best result.

ID	Objectives	$\lambda_{\mathrm{corr}},\lambda_{\mathrm{rank}}$	Normalization	PLCC ↑	SRCC ↑	RMSE ↓
D1	MSE	—	none	0.7618	0.7942	0.0578
D2	MSE	—	EMA	0.7626	0.7948	0.0575
D3	Corr + Rank	0.8, 0.2	none	0.7721	0.7974	0.0538
D4	Corr + Rank	0.8, 0.2	fixed scale	0.7758	0.7998	0.0527
**D5**	**Corr + Rank**	**0.8, 0.2**	**EMA**	**0.7784**	**0.8019**	**0.0519**
D6	Corr + Rank + Var	0.6, 0.2	EMA	0.7749	0.7992	0.0531

**Table 12:** Per-image inference profiling on a single A800 80GB GPU (batch size 1, AMP, no TTA).

Stage	Time (ms)	Share
Image read + 216 patch cropping	35	10.8%
ResNet-50 patch feature extraction	232	71.8%
Linear projection 2048→512	7	2.2%
Hybrid graph construction	18	5.6%
3-layer residual GCN	22	6.8%
Gated attention + regression head	3	0.9%
CUDA/PyTorch overhead	6	1.9%
Total	323	100%

**Table 13:** Backbone ablation: ResNet-50 versus Swin-T (graph, GCN, readout, and loss fixed).

Backbone	Params (M)	MACs (G)	Feature Extraction Time (ms)	PLCC ↑	SRCC ↑	RMSE ↓
ResNet-50	25.61	1153.59	323	0.7784	0.8019	0.0519
Swin-T	30.84	1672.30	412	0.7896	0.8127	0.0506

## Data Availability

The dataset supporting the current study is available online (UHD-IQA Benchmark Database, https://database.mmsp-kn.de/uhd-iqa-benchmark-database.html, accessed on 22 June 2026).

## Abbreviations

The following abbreviations are used in this manuscript:

UHD	Ultra-High Definition
BIQA	Blind Image Quality Assessment
UHD-BIQA	The UHD Blind Image Quality Assessment task
GLUQ	Global-Local representation learning for UHD image Quality assessment
UHD-IQA	The UHD Image Quality Assessment database
PLCC	Pearson Linear Correlation Coefficient
SRCC	Spearman Rank-Order Correlation Coefficient
RMSE	Root Mean Square Error
GRL	Graph Representation Learning
KNN	*k*-Nearest Neighbor
GCN	Graph Convolutional Network
EMA	Exponential Moving Average
TTA	Test-Time Augmentation
MLP	Multi-Layer Perceptron
MOS	Mean Opinion Score
MAC	Multiply–Accumulate

## Appendix A. Reference Implementation Sketch

Algorithm A1 summarizes the core inference pipeline of GLUQ at a high level. It can serve as a concise reproducibility checklist when re-implementing the method.

**Algorithm A1** Inference pipeline of GLUQ
**Input:** UHD image I; grid $\left(G_{w},G_{h}\right)$; patch size *P*; neighborhood size *k* **Output:** predicted quality $\hat{y}$ 1. Sample $N=G_{w}G_{h}$ patch centers on an aspect-ratio–aligned grid. 2. Extract patches ${\left\{x_{i}\right\}}_{i=1}^{N}$ of size P×P from I. 3. Encode each patch with a CNN backbone to obtain embeddings $\left\{h_{i}\right\}$. 4. Build the graph using the general hybrid KNN formulation with the selected spatial-feature mixing weight. 5. Apply *L* residual GCN layers to propagate context on the graph. 6. Compute attention weights $\left\{a_{i}\right\}$ and aggregate $z=\sum_{i}a_{i}h_{i}$. 7. Predict $\hat{y}=sf\left(z\right)+b$ with affine calibration.

## References

[1] Yang P., Sturtz J., Qingge L. (2023). Progress in blind image quality assessment: A brief review. Mathematics.

[2] Zhang L., Zhang L., Mou X., Zhang D. (2011). A comprehensive evaluation of full reference image quality assessment algorithms. Proceedings of the IEEE International Conference on Image Processing, Orlando, FL, USA, 30 September–3 October 2012.

[3] Min X., Duan H., Sun W., Zhu Y., Zhai G. (2024). Perceptual video quality assessment: A survey. Sci. China Inf. Sci..

[4] Saad M.A., Bovik A.C., Charrier C. (2012). Blind image quality assessment: A natural scene statistics approach in the DCT domain. IEEE Trans. Image Process..

[5] Narwaria M., Lin W. (2010). Objective image quality assessment based on support vector regression. IEEE Trans. Neural Netw..

[6] Li C., Bovik A.C., Wu X. (2011). Blind image quality assessment using a general regression neural network. IEEE Trans. Neural Netw..

[7] Yu S., Wang J., Gu J., Jin M., Ma Y., Yang L., Li J. (2023). A hybrid indicator for realistic blurred image quality assessment. J. Vis. Commun. Image Represent..

[8] Chen Z., Yu S. (2025). Taylor expansion-based Kolmogorov–Arnold network for blind image quality assessment. J. Vis. Commun. Image Represent..

[9] Yu S., Wu S., Wang L., Jiang F., Xie Y., Li L. (2017). A shallow convolutional neural network for blind image sharpness assessment. PLoS ONE.

[10] Ying Z., Niu H., Gupta P., Mahajan D., Ghadiyaram D., Bovik A.C. (2020). From Patches to Pictures (PaQ-2-PiQ): Mapping the Perceptual Space of Picture Quality. Proceedings of the IEEE/CVF Conference on Computer Vision and Pattern Recognition (CVPR), Seattle, WA, USA, 13–19 June 2020.

[11] Zhang W., Ma K., Yan J., Deng D., Wang Z. (2020). Blind image quality assessment using a deep bilinear convolutional neural network. IEEE Trans. Circuits Syst. Video Technol..

[12] Madhusudana P.C., Birkbeck N., Wang Y., Adsumilli B., Bovik A.C. (2022). Image Quality Assessment Using Contrastive Learning. IEEE Trans. Image Process..

[13] Agnolucci L., Galteri L., Bertini M., Del Bimbo A. (2024). Arniqa: Learning distortion manifold for image quality assessment. Proceedings of the IEEE/CVF Winter Conference on Applications of Computer Vision, Waikoloa, HI, USA, 3–8 January 2024.

[14] You J., Korhonen J. (2021). Transformer for image quality assessment. Proceedings of the IEEE International Conference on Image Processing (ICIP), Anchorage, AK, USA, 19–22 September 2021.

[15] Lee S.-H., Kim S.-W. (2023). Dual-branch vision transformer for blind image quality assessment. J. Vis. Commun. Image Represent..

[16] Kwon D., Kim D., Ki S., Jo Y., Lee H.-E., Kim S.J. (2024). CLIP-Guided Attribute Aware Pretraining for Generalizable Image Quality Assessment. arXiv.

[17] Xiang J., Dang Y., Chen P., Liang R., Lin W. (2026). Learning Decoupled Features with Perceptual Distillation for Blind Image Quality Assessment. IEEE Trans. Image Process..

[18] Li W., Li G., Zhao T., Li C., Sun W., Wang H., Zheng B., Chen C., Min X., Zhai G. (2025). Q-Insight: Understanding Image Quality via Visual Reinforcement Learning. arXiv.

[19] Bi X., He X., Xiong S., Liu Z., Sheriff R.E. (2026). Degradation-aware Contrastive Learning for Blind Image Quality Assessment. IEEE Signal Process. Lett..

[20] Lin J., Cui Y., Fang C., Pan B., Pan C., Jiang G., Zhang S., Ma S., Tian Q. (2026). Multi-modal Cross-Attention Guided Network for Audio-Visual Quality Evaluation via Visual Saliency and Mel-spectrum Features. IEEE Trans. Circuits Syst. Video Technol..

[21] Huang H., Wan Q., Korhonen J. (2024). High Resolution Image Quality Database. arXiv.

[22] Hosu V., Agnolucci L., Iso D., Saupe D. Image Intrinsic Scale Assessment: Bridging the Gap Between Quality and Resolution. Proceedings of the IEEE/CVF International Conference on Computer Vision (ICCV).

[23] Hosu V., Agnolucci L., Wiedemann O., Iso D., Saupe D. (2024). UHD-IQA Benchmark Database: Pushing the Boundaries of Blind Photo Quality Assessment. Computer Vision—ECCV 2024 Workshops, Proceedings, Part IX.

[24] Sun W., Zhang W., Cao Y., Cao L., Jia J., Chen Z., Zhang Z., Min X., Zhai G.-T. (2025). Assessing UHD Image Quality from Aesthetics, Distortions, and Saliency. Computer Vision—ECCV 2024 Workshops, Proceedings.

[25] Hosu V., Conde M.V., Agnolucci L., Barman N., Zadtootaghaj S., Timofte R., Sun W., Zhang W., Cao Y., Cao L. (2025). AIM 2024 Challenge on UHD Blind Photo Quality Assessment. Computer Vision—ECCV 2024 Workshops, Proceedings.

[26] Gu J., Meng Q., Zhao S., Wang Y., Yu S., Sun Q. (2025). Super-Resolved Pseudo Reference in Dual-Branch Embedding for Blind Ultra-High-Definition Image Quality Assessment. Electronics.

[27] Sun S., Yu T., Xu J., Zhou W., Chen Z. (2022). GraphIQA: Learning Distortion Graph Representations for Blind Image Quality Assessment. IEEE Trans. Multimed..

[28] Jia S., Chen B., Li D., Wang S. (2022). No-reference Image Quality Assessment via Non-local Dependency Modeling. Proceedings of the IEEE International Workshop on Multimedia Signal Processing (MMSP), Shanghai, China, 26–28 September 2022.

[29] Wang H., Liu J., Tan H., Lou J., Liu X., Zhou W., Liu H. (2024). Blind Image Quality Assessment via Adaptive Graph Attention. IEEE Trans. Circuits Syst. Video Technol..

[30] Liu Y., Guo J. (2025). Image Quality Assessment by Enabling Inter-Patch Message Passing via Graph Convolutional Networks. Neural Comput. Appl..

[31] Veličković P., Cucurull G., Casanova A., Romero A., Liò P., Bengio Y. Graph Attention Networks. Proceedings of the International Conference on Learning Representations (ICLR).

[32] Xu K., Hu W., Leskovec J., Jegelka S. How Powerful are Graph Neural Networks?. Proceedings of the International Conference on Learning Representations (ICLR).

[33] Khoshraftar S., An A. (2024). A Survey on Graph Representation Learning Methods. ACM Trans. Intell. Syst. Technol..

[34] Kipf T.N., Welling M. Semi-Supervised Classification with Graph Convolutional Networks. Proceedings of the International Conference on Learning Representations (ICLR).

[35] Xu J., Zhou W., Chen Z. (2021). Blind Omnidirectional Image Quality Assessment with Viewport Oriented Graph Convolutional Networks. IEEE Trans. Circuits Syst. Video Technol..

[36] Xie W., Bian T., Wang M. KgMBQA: Quality Knowledge Graph-driven Multimodal Blind Image Assessment. Proceedings of the Thirty-Fourth International Joint Conference on Artificial Intelligence (IJCAI).

[37] Cavoretto R., De Rossi A., Lancellotti S., Romaniello F. (2024). Node-bound communities for partition of unity interpolation on graphs. Appl. Math. Comput..

[38] Cavoretto R., Comoglio C., De Rossi A. (2025). Community detection methods for GBF-PUM signal approximation on graphs. Appl. Math. Comput..

[39] He K., Zhang X., Ren S., Sun J. Deep Residual Learning for Image Recognition. Proceedings of the IEEE Conference on Computer Vision and Pattern Recognition (CVPR).

[40] Deng J., Dong W., Socher R., Li L.-J., Li K., Fei-Fei L. (2009). ImageNet: A Large-Scale Hierarchical Image Database. Proceedings of the 2009 IEEE Conference on Computer Vision and Pattern Recognition (CVPR), Miami, FL, USA, 20–25 June 2009.

[41] Belkin M., Niyogi P. (2003). Laplacian Eigenmaps for Dimensionality Reduction and Data Representation. Neural Comput..

[42] Ilse M., Tomczak J.M., Welling M. Attention-based deep multiple instance learning. Proceedings of the International Conference on Machine Learning (ICML).

[43] Liu Z., Hu H., Lin Y., Yao Z., Xie Z., Wei Y., Ning J., Cao Y., Zhang Z., Dong L. (2022). Swin transformer v2: Scaling up capacity and resolution. Proceedings of the IEEE/CVF Conference on Computer Vision and Pattern Recognition (CVPR), New Orleans, LA, USA, 18–24 June 2022.

